# From incision to implantation: holter-based stress profiling in ophthalmology residents during cataract surgery

**DOI:** 10.1007/s00417-026-07189-1

**Published:** 2026-03-09

**Authors:** Kemal Tekin, Kubra Ozdemir Yalcinsoy, Elcin Ozdemir Tutan, Ayberk Bilir, Mehmet Murat Uzel, Merve Inanc Tekin

**Affiliations:** 1 Ophthalmology Department, University of Health Sciences, Ulucanlar Eye Training and Research Hospital, Kale Street, Number:59, Altindag-Ankara, 06240 Turkey; 2 Ophthalmology Department, Ulucanlar Eye Training and Research Hospital, Ankara, Turkey; 3 Cardiology Department, Yenimahalle Training and Research Hospital, Ankara, Turkey

**Keywords:** Cataract surgery, Heart rate, Holter monitoring, Intraoperative stress, Ophthalmology residents

## Abstract

**Purpose:**

To objectively evaluate intraoperative stress responses in ophthalmology residents by assessing heart rate (HR) and heart rate variability (HRV) during distinct steps of cataract surgery using Holter monitoring.

**Methods:**

In this prospective study, 26 ophthalmology residents performed standard, uncomplicated phacoemulsification surgery while continuously monitored with a 3-channel digital Holter device. Each surgery was divided into four steps: (1) incision and viscoelastic injection, (2) capsulorhexis, (3) nucleus removal, and (4) cortical aspiration with intraocular lens implantation. Time-domain HR parameters including maximum HR, minimum HR, and mean HR values were recorded and analyzed at baseline and during each surgical step. HRV metrics including mean NN intervals, SDNN, SDANN, SDNN index, and RMSSD were also analyzed.

**Results:**

All surgical steps showed significantly elevated mean HR compared to baseline (*p* < 0.001). The highest mean HR was observed during the capsulorhexis step (108.9 ± 7.2 bpm), followed by nucleus removal (102.7 ± 14.0 bpm). HR remained elevated throughout the procedure without returning to baseline, suggesting sustained sympathetic activation. HRV indices revealed moderate autonomic variability, with SDNN (66.5 ± 24.2 ms) and RMSSD (34.9 ± 19.6 ms) indicating heightened arousal with preserved vagal tone. No significant correlation was found between HR/HRV parameters and residency duration or surgical time (*p* > 0.05 for all).

**Conclusions:**

Ophthalmology residents experience sustained autonomic stress throughout all steps of cataract surgery, with the highest sympathetic activation occurring during capsulorhexis step. These findings can underscore the utility of HRV monitoring in surgical training and support the integration of stress management and simulation strategies into ophthalmology residency curricula.

## Key messages

*****What is known***:**

- Cataract surgery is among the most stressful procedures for ophthalmology residents because of its microsurgical precision and steep learning curve.
- Physiological stress during surgery can be objectively assessed through heart rate and heart rate variability measurements.

*****What is new***:**

- This is the first study to use continuous Holter monitoring to objectively quantify stepwise stress responses in ophthalmology residents during cataract surgery.
- The capsulorhexis step elicited the highest heart rate levels, identifying it as the most physiologically stressful phase of the procedure.
- Residents exhibited sustained sympathetic activation throughout the surgery, with no return to baseline between steps.

## Introduction

Cataract surgery is the most commonly performed ophthalmic surgical procedure worldwide. Despite its routine nature, it remains technically complex, requiring precise fine motor control, real-time decision-making, and continuous focus under optical magnification [1, 2]. For ophthalmology residents, particularly those in the early stages of surgical training, cataract surgery can pose significant cognitive and physiological stress, which may affect surgeons’ performance and surgical outcomes [2–4]. This stress might arise from performance anxiety, fear of complications, and the pressure of working under supervision in high-stakes clinical settings.

Mild and tolerable levels of stress may enhance surgical performance by increasing alertness, concentration, focus, and operational efficiency [5, 6]. In contrast, excessive stress is often counterproductive and can adversely impact multiple aspects of both cognitive and technical performance in the surgical setting [7–9]. Stress in the surgical setting has been shown to impact precision, increase error rates, and alter the learning curves of surgeons [7–9]. Traditionally, the evaluation of surgical stress has relied on a few subjective methods, such as questionnaires or observer ratings, which can be prone to bias [10, 11]. In contrast, objective physiological metrics, such as heart rate variability (HRV) and real-time cardiac monitoring, have emerged as reliable indicators of the acute stress response in clinical settings. Holter monitoring, a continuous ambulatory electrocardiographic recording method, offers a non-invasive, objective way to assess sympathetic nervous system activation during surgery by analyzing the HRV [12]. It has been proven that HRV is a reliable objective method for assessing stress in workplace environments [13]. It can identify intraoperative stressors, determine which surgical techniques induce the highest stress levels in surgeons, and reveal differences in stress responses between performing and assisting roles during surgery [12–14].

Previous studies in various surgical fields, particularly in general surgery, have demonstrated that certain steps of surgical procedures are associated with increased physiological stress responses [9, 15–18]. However, such data remain limited in ophthalmology, despite intraocular procedures requiring high precision and mental load. Given the complexity and multi-step structure of cataract surgery, it is plausible that stress levels vary depending on the surgical stage (e.g., incision, capsulorhexis, phacoemulsification, intraocular lens implantation). Identifying which steps are most stressful may guide more focused simulation, supervision, and stress management interventions within residency curricula.

From this perspective, this study aims to objectively evaluate the physiological stress responses of ophthalmology residents during distinct steps of cataract surgery using Holter monitoring. Additionally, by analyzing heart rate variability across each surgical step—from incision to intraocular lens (IOL) implantation—it further seeks to identify the most stress-inducing steps of the procedure.

## Methods

This hospital-based, prospective observational study was conducted among ophthalmology residents at a Tertiary Eye Training and Research Hospital. The study protocol and data collection procedures were approved by the institutional ethics committee (approval number: E-93471371-514.10.10.10.10.10.10–289405062; *Clinical trial number: not applicable*), and conducted in accordance with the principles of the Declaration of Helsinki. All residents were aware of their inclusion in the study, participated voluntarily, and provided informed consent.

Only uncomplicated cataract surgeries that were performed entirely by the ophthalmology residents without any intraoperative complications were included in the study analysis. To standardize the level of surgical difficulty, only cases involving simple cataracts with grade 2–3 nuclear opacities (as classified by the Lens Opacities Classification System III, LOCS III) with adequate pupil dilation were analyzed. Surgeries were excluded if the operated eyes had dense cataracts, intraoperative floppy iris syndrome, poor pupil dilation, pseudoexfoliation syndrome, corneal scarring, zonular defects or phacodonesis, glaucoma, uveitis, narrow anterior chamber angles, high myopia, or poor patient cooperation. Moreover, only cases that were completed by the same resident from the first incision to the final step of IOL implantation were selected for recording and analysis. Surgeries involving supervisor intervention, surgical assistance, or any surgical complications were not evaluated. Additionally, to ensure standardization, residents were advised to refrain from alcohol consumption for 24 h and from smoking or consuming caffeine-containing beverages for at least 6 h prior to the placement of the Holter device. Residents who had known psychiatric disorders or those receiving antiarrhythmic, antihypertensive, or anxiolytic medications were also excluded from the study.

Standard, uncomplicated phacoemulsification surgery was performed in all cases. For the purpose of stress evaluation, the surgical procedure was divided into four distinct steps: *Step 1 – Pre-phacoemulsification step*: This stage involved creating the main and side-port corneal incisions and injecting viscoelastic into the anterior chamber. *Step 2 – Capsulorhexis step*: This stage began with the initiation of the anterior capsulorhexis and concluded upon its completion. *Step 3 – Nucleus removal step*: The nucleus was divided and totally extracted using the stop-and-chop technique. *Step 4 – Cortical removal and intraocular lens implantation step*: This final phase involved aspiration of the cortical material, followed by implantation of a preloaded monofocal 1-piece IOL into the capsular bag. The start and end times of each surgical step were recorded for all cases, enabling phase-specific analysis. All cataract surgeries were performed using the same phacoemulsification system (Alcon Centurion^®^ Vision System, Alcon Laboratories, Fort Worth, TX, USA) and identical surgical instruments for all cases, with no device-related differences between surgeons.

In order to objectively assess the residents’ stress levels during the distinct steps of cataract surgery, a rhythm Holter device was used in this study. The mini 3-channel digital cardiac Holter monitor recorder (BI9800TL+7D, Biomedical Instruments) was attached to the residents just before entering the operating room and was removed promptly after the procedure was completed. An experienced cardiologist (E.O.T.), blinded to the identities and surgical performance of the residents, evaluated the Holter recordings. Prior to analysis, the clinician verified the quality and completeness of each recording. Each predefined step of the phacoemulsification procedure was analyzed individually to identify variations in physiological stress responses that were step-specific.

HRV was measured using the algorithms of the commercial device. Time-domain HRV indices were analyzed using both statistical and geometrical methods. The statistical analysis included the following parameters: Maximum HR, Minimum HR, and Mean HR values were measured both at baseline in the operating room prior to surgery and during each distinct step of the surgery; mean NN (normal to normal intervals); SDNN (standard deviation of all NN intervals); SDANN (standard deviation of the average NN intervals calculated over 5-minute segments); SDNN index (mean of the standard deviations of all 5-minute NN interval segments throughout the entire recording); RMSSD (root mean square of successive differences between normal-to-normal [NN] intervals) [19]. All measurements were performed in accordance with the Task Force of The European Society of Cardiology and The North American Society of Pacing and Electrophysiology [20].

### Statistical analysis

The study data were analyzed using the Statistical Package for the Social Sciences (SPSS) version 22.0 for Windows (SPSS Inc., Chicago, IL). An a priori power analysis was performed to determine the minimum required sample size for detecting a statistically significant difference in physiological stress levels across the surgical steps. Based on previous studies, and assuming a moderate effect size (Cohen’s d = 0.5), a power of 80%, and a significance level (α = 0.05), the required minimum sample size was calculated to be 16 participants. The analysis was conducted using G*Power software. Descriptive statistics were reported as frequencies and percentages for categorical variables, and as means ± standard deviations for continuous variables. The normality of data distribution was assessed using both visual (e.g., histograms, Q–Q plots) and analytical methods (e.g., Shapiro–Wilk test). To compare the mean HR values between different surgical steps, a repeated-measure ANOVA was performed. Comparisons between male and female residents were performed using independent samples t-test with Welch correction or Mann–Whitney U test, as appropriate, based on data distribution. The Bonferroni post hoc test was used to assess the pairwise comparison of the significantly different means when the overall ANOVA model was significant. To evaluate the relationship between residents’ surgical experience and physiological stress parameters, Pearson or Spearman correlation analyses were performed, depending on the distribution of the data. Specifically, correlations were tested between residency duration and operation time with mean HR values and HRV indices obtained from the Holter recordings. A p-value of less than 0.05 was considered statistically significant.

## Results

In this study, 26 uncomplicated phacoemulsification surgeries performed by 26 different ophthalmology residents were analyzed. Among the residents, 17 (65.4%) were female and 9 (34.6%) were male. The mean age of the participants was 26.9 ± 1.35 years (range: 26–32), and the mean duration of their residency training was 23.3 ± 5.3 months (range: 18–39). Prior to study inclusion, participating residents had completed a mean of 120.0 ± 29.3 cataract surgeries (range: 80–182), reflecting a moderate and relatively homogeneous level of surgical experience. The mean duration of the surgeries was 31.6 ± 7.4 min (range: 17–41), and no intraoperative complications were observed during any of the surgeries.

**Fig. 1:**
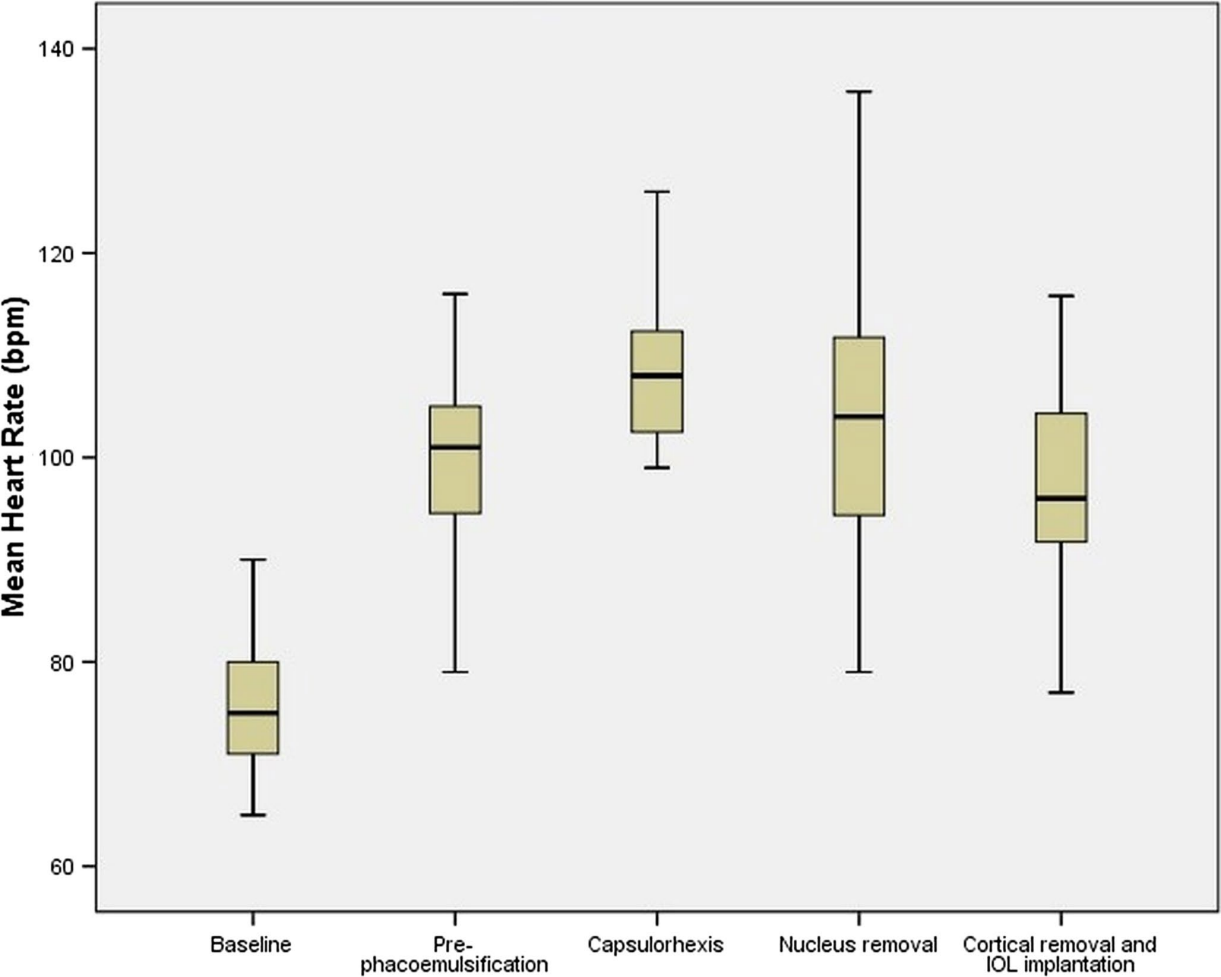
The mean heart rate values at baseline and during each distinct step of the phacoemulsification surgery

The minimum, maximum, and mean HR values at baseline and during each distinct step of the surgery, along with HRV indices obtained by the Holter device, are presented in Table 1. The mean HR values were as follows: 76.4 ± 7.1 beats per minute (bpm) at baseline; 99.4 ± 10.7 bpm at the Step 1 *(Pre-phacoemulsification);* 108.9 ± 7.2 bpm at the Step 2 *(Capsulorhexis);* 102.7 ± 14.0 bpm at the Step 3 *(Nucleus removal);* and 96.6 ± 11.11 bpm at the Step 4 *(Cortical removal and IOL implantation)* (Fig. 1). The mean HR values during each distinct step of the surgery were statistically significantly higher compared to the baseline values (*p* < 0.05 for each). All participants demonstrated an increase in mean HR compared with baseline during at least one surgical step. The HRV indices recorded by the Holter monitor revealed the following results: the mean NN interval was 611.5 ± 75.3 ms, SDNN was 66.5 ± 24.2 ms, SDANN was 39.1 ± 27.8 ms, the SDNN index was 53.6 ± 14.5 ms, and RMSSD was 34.9 ± 19.6 ms. These HRV values reflect both overall autonomic nervous system activity and short-term HRV fluctuations observed during the surgical procedure.

**Table 1:** Baseline and intraoperative heart rate values and heart rate variability indices recorded by the Holter device

Parameters	Mean ± SD (Range)
**Heart Rate Values** *(bpm)*	
Minimum heart rate at Baseline	67.7 ± 6.5 (60–82)
Maximum heart rate at Baseline	85.0 ± 8.8 (69–98)
Mean heart rate at Baseline	76.4 ± 7.1 (65–90)
Minimum heart rate at Step 1	81.3 ± 11.4 (60–106)
Maximum heart rate at Step 1	116.5 ± 13.2 (83–139)
Mean heart rate at Step 1	99.4 ± 10.7 (78–116)
Minimum heart rate at Step 2	91.6 ± 9.0 (73–112)
Maximum heart rate at Step 2	123.9 ± 8.7 (110–144)
Mean heart rate at Step 2	108.9 ± 7.2 (99–126)
Minimum heart rate at Step 3	80.6 ± 10.5 (59–97)
Maximum heart rate at Step 3	118.4 ± 14.7 (89–156)
Mean heart rate at Step 3	102.7 ± 14.0 (79–135)
Minimum heart rate at Step 4	79.6 ± 9.2 (60–95)
Maximum heart rate at Step 4	111.8 ± 12.4 (90–131)
Mean heart rate at Step 4	96.6 ± 11.11 (77–115)
**Heart Rate Variability Indices** *(ms)*	
Mean NN	611.5 ± 75.3 (490–815)
SDNN	66.5 ± 24.2 (35–146)
SDANN	39.1 ± 27.8 (15–143)
SDNN Index	53.6 ± 14.5 (30–83)
RMSSD	34.9 ± 19.6 (14–91)

*Bpm: beats per minute*,* NN: Normal to Normal; SDNN: Standard deviation of all NN; SDANN: Standard deviation of average NN; RMSSD: Root mean square of successive differences.*

Step 1 indicates *Pre-phacoemulsification*

Step 2 indicates *Capsulorhexis*

Step 3 indicates *Nucleus removal*

Step 4 indicates *Cortical removal and IOL implantation*

The comparison of minimum, maximum, and mean HR values across the four surgical steps is demonstrated in Table 2. The mean HR during step 2 (Capsulorhexis) was statistically significantly higher compared to the other three surgical steps (*p* < 0.05, for all). Although the mean HR during step 3 (Nucleus removal) was also higher compared to step 1 (Pre-phacoemulsification) and step 4 (Cortical removal and IOL implantation), these differences did not reach statistical significance in pairwise comparisons (*p* > 0.05, for all). Furthermore, there were no statistically significant differences in minimum or maximum HR values between the four surgical steps (*p* = 0.086 and *p* = 0.200, respectively).

**Table 2:** Comparison of Minimum, Maximum, and Mean heart rate values across distinct steps of the surgery

Heart Rate Values	Step 1	Step 2	Step 3	Step 4	*P*
**Minimum HR** *(bpm)* mean ± SD (range)	81.3 ± 11.4 (60–106)	91.6 ± 9.0 (73–112)	80.6 ± 10.5 (59–97)	79.6 ± 9.2 (60–95)	0.086^¥^
**Maximum HR** *(bpm)* mean ± SD (range)	116.5 ± 13.2 (83–139)	123.9 ± 8.7 (110–144)	118.4 ± 14.7 (89–156)	111.8 ± 12.4 (90–131)	0.200^¥^
**Mean HR** *(bpm)* mean ± SD (range)	99.4 ± 10.7 (78–116)	108.9 ± 7.2 (99–126)	102.7 ± 14.0 (79–135)	96.6 ± 11.11 (77–115)	**0.002** ^**¥**^ **0.001**^**a**^, 0.157^b^, 0.178^c^, **0.011**^**d**^, < **0.001**^**e**^, 0.073^f^

*Bpm: beats per minute*,* HR: Heart rate.*

^¥^Repeated measure of ANOVA (comparison among four groups)

^a^ Significance between Step 1 and Step 2 (pairwise comparison)

^b^ Significance between Step 1 and Step 3 (pairwise comparison)

^c^ Significance between Step 1 and Step 4 (pairwise comparison)

^d^ Significance between Step 2 and Step 3 (pairwise comparison)

^e^ Significance between Step 2 and Step 4 (pairwise comparison)

^f^ Significance between Step 3 and Step 4 (pairwise comparison)

Step 1 indicates *Pre-phacoemulsification*

Step 2 indicates *Capsulorhexis*

Step 3 indicates *Nucleus removal*

Step 4 indicates *Cortical removal and IOL implantation*

***Bold values indicate statistically significant values.***

The comparison of intraoperative HR and HRV parameters between male and female residents is revealed in Table 3. Subgroup analysis according to gender showed no statistically significant differences in baseline HR, step-specific HR, or HRV parameters between male and female residents (*p* > 0.05 for all).

**Table 3:** Comparison of intraoperative heart rate and heart rate variability between male and female residents

Parameters	Female (*n* = 17) Mean ± SD	Male (*n* = 9) Mean ± SD	*P*
Mean heart rate at Baseline	75.4 ± 6.9	79.3 ± 7.6	0.390^**§**^
Mean heart rate at Step 1	98.5 ± 12.0	102.1 ± 8.7	0.443^**§**^
Mean heart rate at Step 2	111.4 ± 8.0	104.0 ± 6.5	0.153^**§**^
Mean heart rate at Step 3	105.4 ± 15.2	98.0 ± 10.5	0.199^**§**^
Mean heart rate at Step 4	96.3 ± 12.0	97.4 ± 9.1	0.821^**§**^
Mean NN (ms)	604.7 ± 68.9	621.9 ± 81.4	0.101^**§**^
SDNN (ms)	64.9 ± 22.4	69.9 ± 26.8	0.256^**§**^
SDANN (ms)	41.6 ± 25.5	35.7 ± 29.0	0.305^**§**^
SDNN index (ms)	51.0 ± 15.5	56.6 ± 13.2	0.459^**§**^
RMSSD (ms)	33.1 ± 18.5	36.4 ± 21.6	0.705^**§**^

*Bpm: beats per minute*,* NN: Normal to Normal; SDNN: Standard deviation of all NN; SDANN: Standard deviation of average NN; RMSSD: Root mean square of successive differences.*

^**§**^Mann–Whitney U test

Step 1 indicates *Pre-phacoemulsification*

Step 2 indicates *Capsulorhexis*

Step 3 indicates *Nucleus removal*

Step 4 indicates *Cortical removal and IOL implantation*

Additionally, correlation analysis revealed no statistically significant relationships between the duration of residency training and operation time with any of the HR values and HRV indices obtained from the Holter recordings (*p* > 0.05, for all).

## Discussion

This study provides a stepwise physiological evaluation of intraoperative stress on ophthalmology residents during phacoemulsification surgery. By analyzing HR values across distinct steps of surgery and assessing HRV indices using continuous Holter monitoring, autonomic responses during four defined steps were objectively measured and compared with baseline values. One of the key findings of the study was that the HR values increased significantly during all surgical steps compared to baseline, indicating a consistent activation of the autonomic stress response throughout the surgery. These data also exhibit a persistent, stage-dependent sympathetic activation, with no step showing a return to baseline HR. Given the short and consecutive nature of the surgical steps in cataract surgery, it is important to note that the absence of heart rate recovery between steps likely reflects a cumulative and sustained sympathetic activation rather than isolated, step-specific stress responses. This may highlight the sustained psychological and physiological burden placed on residents during phacoemulsification. Another important finding was that the capsulorhexis step elicited the highest HR, indicating it was the most stressful part of the surgery. This observation is in accordance with the complexity and precision required for creating a continuous curvilinear capsulorhexis. Capsulorhexis is foundational for performing a successful cataract surgery, and inadequate and inappropriate capsulorhexis formation can adversely affect the following surgical steps and increase the risk of intraoperative and postoperative complications, such as radial tears, zonular or posterior capsule rupture, insufficient capsular support for IOL implantation, and postoperative capsular phimosis, IOL decentration, and posterior capsular opacification [21, 22]. The elevated HR during this step is therefore consistent with heightened anticipatory stress and performance anxiety, both common in surgical training environments [15]. Furthermore, it was also found that even the initial incision step (Step 1) was associated with a sharp HR increase (~ 23 bpm above baseline), which might indicate preoperative anticipatory stress, possibly related to fear of making the first incision under supervision or initiating a complex task. Similarly, the elevated HRs during nucleus removal and IOL implantation demonstrate that residents remained under a sustained sympathetic drive throughout the operation, without autonomic return to the resting state at any point. These findings suggest that residents experience the entire surgical timeline as a high-stakes continuum, rather than assuming that stress peaks only at the beginning or during the most technically challenging step.

The influence of surgical experience and case complexity on intraoperative stress has been highlighted in several recent studies. Kaushik et al. [23] reported that surgeons exhibited significantly higher HR and blood pressure during complicated cataract procedures compared with routine uncomplicated cases, indicating that case complexity independently elevates physiological stress, even among experienced surgeons. Additionally, Cap et al. [24] introduced a normalized HRV-derived stress index to quantify acute intraoperative stress and demonstrated that less experienced cataract surgeons had higher HRV stress indices compared with those with greater surgical volume, with stress decreasing logarithmically as experience increased up to a plateau. These findings align with the notion that case complexity and operative difficulty can override protective effects of experience, emphasizing the dynamic nature of stress responses in the clinical setting. In our cohort, by including only residents with moderate and comparable experience levels, the observed variations in HR and HRV across procedural steps are therefore more likely attributable to task-related stressors and surgical complexity rather than baseline proficiency differences, underscoring the value of objective monitoring approaches capable of capturing these nuanced physiological responses.

Although neuroendocrine biomarkers were not directly assessed in this study, previous research provides important insights into the physiological mechanisms underlying intraoperative stress responses [25]. Cataract surgery has been shown to induce postoperative increases in serum cortisol and urinary catecholamines, reflecting activation of both the hypothalamic–pituitary–adrenal axis and the sympathetic–adrenomedullary system. Importantly, stress processing differs according to surgical experience. Trainees tend to exhibit disproportionately higher adrenaline responses, which are associated with emotional arousal, anxiety, tachycardia, and exaggerated cardiovascular activation [25]. Excessive adrenaline release may impair fine motor control and visuomotor precision—critical elements in microsurgical procedures such as cataract surgery. In contrast, experienced surgeons demonstrate relatively greater noradrenaline-dominant responses, which are linked to focused attention, sustained vigilance, and more controlled autonomic activation [25]. Cortisol elevations, on the other hand, reflect cumulative stress load and prolonged cognitive demand, complementing moment-to-moment autonomic fluctuations captured by HR and HRV. Within this framework, the HR and HRV patterns observed in our resident cohort may represent a predominantly adrenergic stress profile, consistent with heightened emotional and cognitive load during surgical training.

The observed cardiovascular and autonomic stress responses may also be interpreted within the framework of the Yerkes–Dodson law, which describes a non-linear relationship between stress intensity and performance [26, 27]. Moderate stress levels—characterized by controlled elevations in HR, relatively preserved HRV, and balanced autonomic activation—may enhance alertness, concentration, and task engagement. In contrast, excessive stress, reflected by pronounced HR increases, reduced HRV, elevated cortisol levels, and adrenaline-dominant responses, has been associated with impaired fine motor control, decision-making, and situational awareness. This conceptual model helps explain why similar intraoperative stressors may facilitate performance in experienced surgeons, who tend to exhibit more regulated stress responses, but may adversely affect outcomes in trainees, particularly during high-risk or technically demanding phases of surgery. Within this context, the sustained sympathetic activation observed in our resident cohort suggests a stress profile that may approach the upper, potentially performance-limiting range of this curve during certain surgical steps.

Recent evidence indicates that greater surgical experience is associated with reduced hand tremor and improved instrument handling during microsurgical tasks, reflecting adaptive neuromuscular control and autonomic regulation. Specifically in ophthalmology, proficiency has been associated with more stable forceps motion and handling characteristics during delicate procedures [28].

Because hand tremor is closely linked to sympathetic nervous system activation, including increases in HR, these findings provide functional context to the present results. Although physiological tremor was not directly assessed here, the sustained HR elevations observed among residents may represent an underlying physiological substrate associated with stress-mediated motor instability during challenging surgical steps. This perspective supports the notion that HR monitoring may serve as a practical surrogate marker for stress-related motor effects in microsurgical training environments.

In addition to significant elevations in HR values, HRV analysis during the surgery offered complementary insights into the residents’ autonomic responses. The mean NN interval (611.5 ms) corresponded to a mildly elevated heart rate (~ 98 bpm), indicative of basal sympathetic activation—likely driven by cognitive load, performance anxiety, and error anticipation. The SDNN (66.5 ms) and SDNN index (53.6 ms) pointed to preserved autonomic flexibility and short-term variability, while the relatively low SDANN (39.1 ms) suggested sustained sympathetic arousal, possibly due to continuous intraoperative demands. Furthermore, the RMSSD (34.9 ms), a marker of vagal tone, remained within a moderate range, indicating that parasympathetic function was maintained despite procedural stress. Overall, these findings indicate a moderate but physiologically adaptive stress response, supporting the utility of HRV as an objective measure of intraoperative stress and autonomic resilience in surgical training environments.

Only a few studies investigated the challenges and stress levels associated with different steps of cataract surgery among ophthalmology residents. Dooley and O’Brien assessed the relative difficulty of each step of phacoemulsification surgery performed by first- and second-year ophthalmology residents using a questionnaire with a 5-point rating scale [29]. They concluded that capsulorhexis is the most challenging step overall, and first-year residents rated it as more difficult than phacoemulsification, whereas second-year trainees found phacoemulsification more difficult [29]. Prakash et al. [30] also evaluated the perceived difficulty of routine cataract surgery steps among second- and third-year residents by using a questionnaire and reported that the most difficult step for second-year residents was loading a foldable IOL, while for third-year residents it was nuclear emulsification. The differences between their findings and those of the present study may be attributed to several factors: first, their study relied on a subjective assessment method rather than objective physiological measurements; second, residents may have perceived nuclear emulsification as the most difficult step simply because it is the longest part of the procedure; and third, trypan blue dye was routinely used for staining the anterior capsule in their resident cases. Moreover, their study also included mature and hypermature cataracts, which typically require greater phacoemulsification power and complex manipulations. Additionally, in our study, preloaded IOLs were used, and residents did not participate in the IOL loading process, which may explain why this step was not considered stressful. In another study, Rali et al. [31] investigated the stress levels of postgraduate third- and fourth-year ophthalmology residents, analyzing HR with a chest-strap Bluetooth monitor device during each step of cataract surgery. They concluded that quadrant removal, nucleus disassembly, and incision creation are the three most stressful steps for residents [31]. However, they also included all cataract types without standardizing case complexity or cataract density, potentially confounding stress levels across different steps, and the inclusion of more senior residents may have skewed their data, as experienced trainees tend to perform capsulorhexis with more confidence, possibly underestimating the stress associated with this critical step.

The results of this current study are consistent with previous research in other surgical fields and training models, which have shown that increased HR is a reliable real-time marker of intraoperative stress, particularly in the presence of cognitive load, risk of error, or supervisory pressure [13, 32, 33]. However, compared to other surgical fields of medicine, ophthalmic microsurgery adds unique stressors owing to the fine-motor control, which has minimal tolerance for tremor and hesitation, and a high-precision environment under the microscope, which might explain the sustained HR elevation observed across all surgical steps. The sustained increase in HR across all steps of cataract surgery suggests a need to address intraoperative stress more holistically in residency programs, rather than focusing solely on specific steps, such as capsulorhexis. Integrating HR monitoring tools to wet-lab simulations could help residents gain insight into their own stress physiology and develop strategies for autonomic self-regulation. Beyond characterizing intraoperative stress responses, the findings of the present study have important implications for surgical training, patient safety, and surgeon well-being. Objective assessment of physiological stress using HR and HRV monitoring enables the identification of high-stress phases during cataract surgery, facilitates differentiation between experience-related and task-specific stressors, and may allow early recognition of maladaptive stress responses [24]. In particular, the consistently elevated HR observed during capsulorhexis highlights this step as a potential target for focused simulation-based training and graduated autonomy. Incorporating objective stress monitoring into residency curricula may help tailor educational strategies, optimize supervision, and enhance patient safety. Furthermore, stress-modulation approaches such as simulation training, structured feedback, music therapy, and stress-management interventions may support the development of more performance-adaptive stress profiles, potentially protecting surgeons from long-term occupational stress and burnout [34].

This study had some limitations. First, although power analysis showed that it was adequately powered, larger multi-center studies would be needed for broader generalizability of the results. Second, HR and HRV are indirect proxies of psychological stress and do not fully account for subjective perceptions or coping styles, which may be evaluated together with validated stress questionnaires in further studies. Finally, no residents experienced intraoperative complications; therefore, the true peak of HR response under crisis conditions may be underestimated.

In conclusion, this study demonstrates that ophthalmology residents experience elevated HRs throughout all steps of cataract surgery, reflecting a sustained and physiologically appropriate stress response. The capsulorhexis step remains the most demanding from a cardiovascular standpoint, but elevated HR across all phases suggests a persistent state of autonomic arousal during resident-performed cataract surgery. These findings emphasize the value of HR monitoring as an objective stress metric and support the implementation of stress-responsive simulation and training strategies in ophthalmic education.

## Data Availability

The data that support the findings of this study are available from the corresponding author upon reasonable request.
